# MicroRNA-15a-5p Regulates the Development of Osteoarthritis by Targeting PTHrP in Chondrocytes

**DOI:** 10.1155/2019/3904923

**Published:** 2019-03-05

**Authors:** Zhi-xi Duan, Peng Huang, Chao Tu, Qing Liu, Shuang-qing Li, Ze-ling Long, Zhi-hong Li

**Affiliations:** ^1^Department of Orthopedics, The Second Xiangya Hospital, Central South University, 139 Renmin Road, Changsha 410011, China; ^2^Department of General Surgery, Xiangya Hospital, Central South University, No. 87 Xiangya Road, Changsha 410008, China

## Abstract

**Background and Aims:**

A growing body of research has demonstrated that the degeneration of chondrocytes is the primary cause of osteoarthritis (OA). Parathyroid hormone-related protein (PTHrP) can alleviate the degeneration of chondrocytes via promotion of chondrocyte proliferation and inhibition of terminal differentiation, but the underlying mechanism remains unknown. This study aimed to identify the microRNAs (miRNAs) that may target PTHrP and regulate the proliferation and terminal differentiation of chondrocytes.

**Methods:**

Bioinformatic analysis was used to predict which miRNAs target PTHrP. We collected human knee cartilage specimens to acquire the primary chondrocytes, which we then used to test the expression and function of the targeted miRNAs. To explore the effects of miR-15a-5p on the putative binding sites, specific mimics or inhibitors were transfected into the chondrocytes. Furthermore, a dual-luciferase reporter gene assay and chondrocyte degeneration-related factors were used to verify the possible mechanism.

**Results:**

The expression of PTHrP was upregulated in the OA chondrocytes, whilst miR-15a-5p was downregulated in the OA chondrocytes. A negative correlation was observed between PTHrP and miR-15a-5p. The knockdown of miR-15a-5p promoted the growth of chondrocytes and inhibited calcium deposition, whilst overexpression of miR-15a-5p reversed this trend. The effect of miR-15a-5p overexpression was neutralised by PTHrP. Dual-luciferase reporter assays revealed that PTHrP can be used as a novel targeting molecule for miR-15a-5p.

**Conclusions:**

miR-15a-5p promotes the degeneration of chondrocytes by targeting PTHrP and, in addition to helping us understand the development of OA, may be a potential biomarker of OA.

## 1. Introduction

Osteoarthritis (OA), a degenerative joint condition, is the most common disease in adults and, in addition to leading to high medical expenses, is one of the most frequent causes of pain, loss of function, and psychological disability [1, 2]. Several treatment options are available for relief of OA symptoms, but because the pathogenesis of OA remains unclear, few practical ways of stopping the progressive degradation of the articular cartilage have been found [3, 4].

Deterioration of the smoothness of the articular cartilage is the chief characteristic of OA, and chondrocytes are the only type of cells in the articular cartilage, so dysregulation of cell proliferation and differentiation in chondrocytes contributes directly to OA [5–7]. The primary function of chondrocytes is to maintain the balance of the extracellular matrix, which consists mainly of collagen and proteoglycans. However, the pattern of gene expression and the related mechanisms involved in chondrocyte action remain unclear.

Parathyroid hormone-related protein (PTHrP, also known as PTHLH) is a widely occurring protein produced by most tissues in the body [8, 9] and is an essential factor in many of the physiological and pathological processes involved in OA [10]. Numerous reports have confirmed that PTHrP contributes to chondrocyte proliferation and inhibits terminal differentiation [8, 11–13]. However, the regulation of PTHrP in OA is still poorly understood.

MicroRNAs (miRNAs), about 23 nt of endogenous small noncoding RNA, are involved in the regulation of target protein-encoding genes via translational inhibition and/or degradation of the target mRNA [14–16]. The expression of various small RNAs in OA changes in relation to that in normal cartilage, which suggests that the expression of small RNA may affect cartilage homeostasis [17]. The significance of miR-15a-5p for the survival and metastasis of tumours, especially during the process of cell proliferation or differentiation, has attracted much attention [18, 19]. The function of miR-15a-5p in regulating the degradation of the cartilage matrix has also been investigated [20]. Unfortunately, the underlying mechanism of the involvement of miR-15a-5p in the progress of OA remains unclear. In this study, we found that PTHrP served as the target of miR-15a-5p and clarified the effects of miR-15a-5p on the proliferation and differentiation of chondrocytes.

## 2. Materials and Methods

### 2.1. Human Specimens

In this study, rheumatoid arthritis and septic arthritis were excluded, and samples of human articular cartilage were obtained from 16 patients with OA (OA Grade III–IV) who underwent total knee arthroplasty. Eight patients with osteosarcoma who were undergoing segmental resection and artificial knee prosthesis replacement or amputation were also recruited to allow collection of normal articular cartilage specimens.  Table 1 lists the corresponding information. Our work was approved by the ethics committee of Second Xiangya Hospital, Central South University (Changsha, Hunan, China).

### 2.2. Isolation and Culture of Chondrocytes

The cartilage specimens were collected immediately after surgery, placed in Petri dishes, and wetted with phosphate-buffered saline solution (PBS). The cartilage samples were cut into 1–3 mm^3^ pieces with two sterile scalpels. The PBS was removed, and the diced tissues were placed in Dulbecco's modified Eagle's medium (DMEM; Sangon, Shanghai, China) with 2.0 mg/mL collagenase type II (Worthington, USA). The tube was capped, covered with Parafilm, and then placed on an orbital shaker at 200 rpm for 10–12 h at 37°C. The next day, the suspension was filtered through a 40-*µ*m mesh into a sterile 50-ml centrifuge tube. The digestate was centrifuged at 1200 rpm for 10 min. The supernatant was removed, and the pellet was gently resuspended in 10 mL of DMEM. The specific protocol of the primary chondrocyte culture is based on the literature [21, 22]. The cells were centrifuged, washed, and resuspended in the medium, placed in a T-25 flask, and cultured in DMEM plus 10% FBS at 37°C, 5% CO_2_. The chondrocytes in the second passage were used in all of the experiments.

### 2.3. Cartilage Tissue and Chondrocyte Staining

The excised cartilage tissues were fixed in 4% paraformaldehyde (PFA), briefly decalcified in 10% ethylenediaminetetraacetic acid (EDTA) for 4 weeks, and sectioned into 4–5 mm thick slices with a scalpel blade. Each slice was embedded in paraffin and sectioned at 6 *µ*m. Serial sections from each slice were stained routinely with Safranin O/Fast Green. The cell-attached slides were washed using PBS and stained using Toluidine blue for 15 min. The cells were rewashed with PBS, and the morphology of the cartilage tissues and cells was assessed.

### 2.4. Immunohistochemical Analysis

After being seeded into special chamber slides, the human primary chondrocytes were fixed with 4% PFA for 10 min, permeabilised with 0.1% Triton X-100 for 15 min, and treated with 1% bovine serum albumin for 30 min. Next, the cells were incubated with PTHrP antibody for 1 h, and the anti-goat IgG antibody conjugated with peroxidase was then incubated for 30 min. Finally, the nuclei were counterstained with haematoxylin. All of the procedures were performed at room temperature.

### 2.5. Calcium Deposition Assay

To evaluate the extent of the calcification or calcium deposition in the intervention and control groups of the cultured primary chondrocytes, we used Alizarin Red S staining, which is one of the most commonly used methods. The cells were fixed at room temperature with 4% PFA for 10 min and exposed to Alizarin Red S for 15 min. The cells were then washed with PBS and then photographed under a microscope.

### 2.6. Western Blotting Analysis

The total protein was extracted from each intervention group by lysing the cells in a radioimmunoprecipitation assay buffer (Beyotime, Shanghai, China). Quantitative analysis of the protein concentrations was performed with a special analytical kit (Beyotime, Shanghai, China). The same amount of proteins was obtained by 10% sodium dodecyl sulphate-polyacrylamide gel electrophoresis and electrotransferred onto polyvinylidene fluoride (PVDF) membranes. The PVDF membranes were then blocked with 5% skimmed milk powder in TBST (Tris-buffered saline solution containing 0.1% Tween 20) and cultured with primary and secondary antibodies. The specific protocol of the western blotting is based on the literature [23]. After washing, the staining was visualised with an enhanced chemiluminescence kit (Beyotime, Shanghai, China), and the specific strip was measured with the MicroChemi 4.2 system (DNR Bio-Imaging Systems, Jerusalem, Israel).

### 2.7. Cell Transfection and Cell Proliferation Analysis

OA chondrocytes in logarithmic phase were seeded into six-well plates and cultured for 24 h. The cells were then allotted to several groups: the siPTHrP group, the negative control (NC) group, the miR-15a-5p mimics group, the NC inhibitors group, and the miR-15a-5p inhibitors group. The plasmids were purchased from GenePharma Co Ltd (Shanghai, China). The cells were transiently transfected with plasmids using the Lipofectamine 2000 reagent (Thermo Fisher Scientific) as previously reported [24]. The corresponding sequences are listed in Table 2. A Cell Counting Kit-8 (CCK-8, Beyotime Biotechnology, China) was used to test cell proliferation after the cells had been incubated for various periods of time. The OD values at 450 nm were measured by spectrophotometry.

### 2.8. Luciferase Reporter Assay

We constructed wild-type and mutant plasmids for PTHrP to explore the targeting link between miR-15a-5p and PTHrP. Table 2 lists the sequences used. We verified the recombinant plasmids via restriction enzyme digestion and DNA sequencing.

HEK293T cells were added into a 12-well plate to confirm the direct interaction between miR-15a-5p and PTHrP mRNA. The cells were transfected with miR-15a-5p mimics and PTHrP wild-type or PTHrP mutants using the Lipofectamine 2000 reagent after 24 hours. The samples were then cotransfected with a pRL-TK plasmid that expresses Renilla luciferase. About 48 hours after the transfection, the cells were lysed and the Firefly and Renilla luciferase activities were detected with a dual luciferase reporter assay system (Biotek, Winooski, VT). The transfection was performed in triplicate.

### 2.9. Quantitative Real-Time PCR (qRT-PCR)

The total RNA was separated by Trizol Reagent (Thermo Fisher Science), reverse transcribed into cDNA, and used for the qRT-PCR analysis, as previously reported [25]. The ABI Prism VIIA7 system was used to quantify the gene expression by qRT-PCR (ABI Prism ViiA7, Applied Biosystems, CA). The level of PTHrP was analysed using qRT-PCR and normalised to actin. The miR-15a-5p was normalised to U6. The primer design of miR-15a-5p was acquired from Sangon (Shanghai, China). The corresponding primer sequences are listed in Table 2.

### 2.10. Statistical Analysis

The statistical analysis was performed using GraphPad Prism Software (Version 7.0). The results are expressed as mean ± standard deviation (SD). Student's *t*-test was used to detect the differences between the two groups. A *P* value of less than 0.05 was considered to indicate significant variation between groups.

## 3. Results

### 3.1. Upregulation of PTHrP in Chondrocytes from Human Knee OA

**Figure 1:**
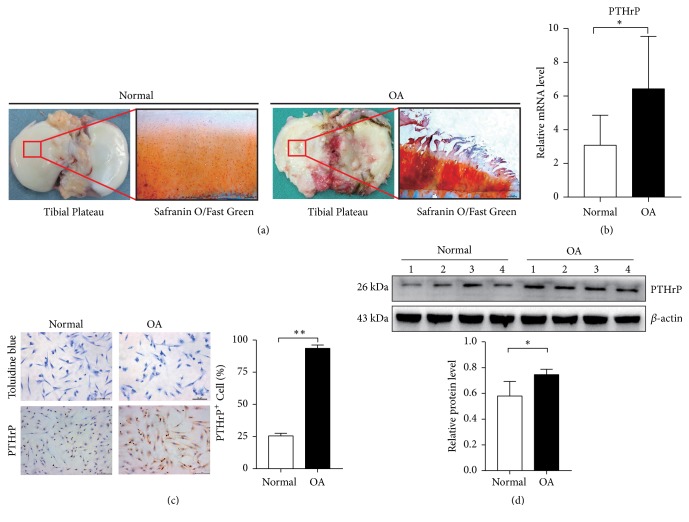
*Expression of PTHrP in human normal and OA chondrocytes*. Human knee articular cartilage was taken from subjects after total knee arthroplasty or amputation surgery. (a) Tissue morphology and Safranin O/Fast Green staining of normal and OA articular cartilage (scale bars, 100 *μ*m). (b) RT-PCR was used to detect the relative levels of PTHrP in normal and OA chondrocytes, *∗p*<0.05, n(normal) = 8, and n(OA) = 16. (c) Toluidine blue staining and immunohistochemical staining of chondrocytes (scale bars, 100 *μ*m). The number of PTHrP (+) cells after IHC is presented as mean ± SD in the lower panels (*∗∗p*<0.01, n = 6). (d) Western blot analysis shows the relative expression of PTHrP in chondrocytes. The quantitative results are presented as mean ± SD in the lower panels (*∗p*<0.05, n = 4).

The general morphology of the normal and OA articular cartilage samples was examined after Safranin O/Fast Green staining, which revealed differences in the surface of the tibial plateaus. The normal articular cartilage was smooth, whilst the OA articular cartilage showed extensive wear (Figure 1(a)). To better understand the related mechanism, the expression of PTHrP was detected by RT-PCR in 8 normal chondrocytes and 16 OA chondrocytes. The results showed a significant increase in the expression of PTHrP in the OA chondrocytes (Figure 1(b)). Furthermore, we cultured chondrocytes from the articular cartilage and observed the cell morphology after Toluidine blue staining (Figure 1(c)). A previous study showed that the level of PTHrP was higher in OA than in normal human knee articular cartilage [26]. Similarly, in this study, immunohistochemical analysis revealed a significantly higher level of PTHrP in the OA chondrocytes than in the normal chondrocytes (*p*<0.01) (Figure 1(c)). Analysis of the PTHrP protein expression in the chondrocytes by western blotting confirmed significant upregulation of PTHrP in the OA chondrocytes* (p*<0.05) (Figure 1(d)).

### 3.2. PTHrP Prevents Terminal Differentiation and Promotes Proliferation in Human OA Chondrocytes

**Figure 2:**
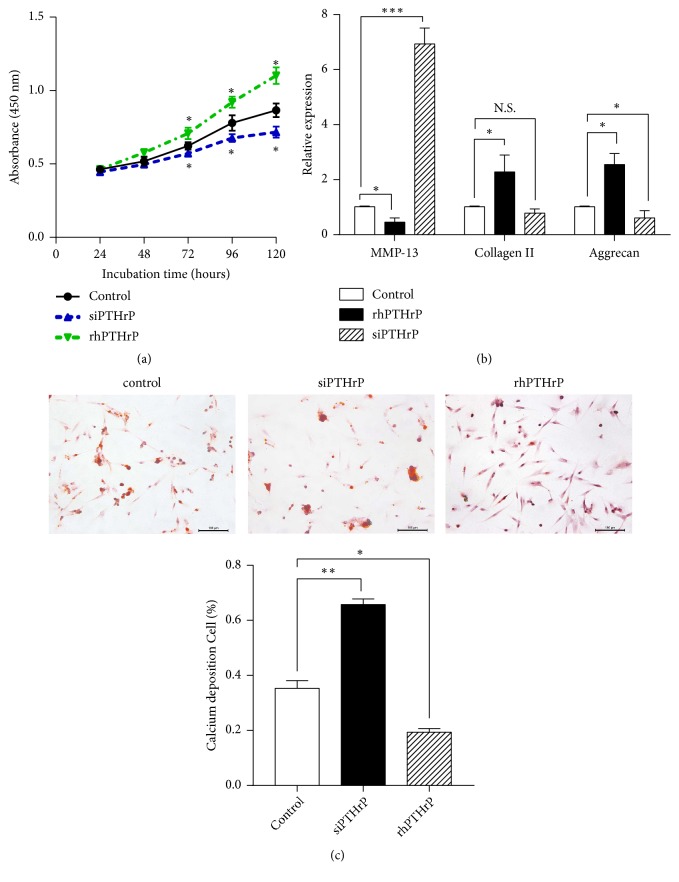
*Effect of PTHrP on chondrocyte proliferation and differentiation*. OA chondrocytes were transfected with siPTHrP or a control plasmid or treated with rhPTHrP. (a) Cell viability was tested with a CCK8 kit after 24, 48, 72, 96, and 120 h. (b) qRT-PCR was performed to examine the MMP-13, collagen II, and aggrecan expression in the chondrocytes (*∗p*<0.05, *∗∗∗p*<0.001 in comparison to the control, n = 6). (c) Calcium deposition was visualised by Alizarin Red S staining (scale bars, 100 *μ*m). Calcium deposition (+) cells are presented as mean ± SD in the lower panels (*∗p*<0.05, *∗∗p*<0.01, and n = 6).

To determine the role played by PTHrP in promoting proliferation and preventing terminal differentiation in human knee OA chondrocytes, cells were cultured with siPTHrP, the corresponding controls, or recombinant human PTHrP (rhPTHrP). As shown in Figure 2(a), a CCK8 experiment showed that the growth rate of primary cultured chondrocytes transfected with siPTHrP was slower than that of the control group, whilst rhPTHrP caused a significant increase in growth (*p<*0.05). qRT-PCR was performed to further test the effect of PTHrP on the chondrocytes. Biomarkers of proliferation and terminal differentiation, including collagen II, aggrecan, and MMP-13, were investigated. MMP13 is a major enzyme that targets cartilage and leads to the degradation of collagen II and proteoglycan. Aggrecan, a proteoglycan, and collagen II are critical components of the cartilage structure and function of chondrocytes [27]. As shown in Figure 2(b), a significant increase of MMP-13 was observed in the siPTHrP group relative to the control group (*p<*0.01), whilst the level of MMP-13 was lower in the rhPTHrP group than in the control group (*p<*0.05). In contrast, the level of collagen II increased in the rhPTHrP group (*p<*0.05, versus the NC group) and decreased in the siPTHrP group in comparison with the NC group (*p<*0.05). The level of aggrecan was also higher in the rhPTHrP group than in the NC group (*p<*0.05) and lower in the siPTHrP group than in the NC group (*p<*0.05).

Alizarin Red S staining demonstrated the presence of calcium deposits in the OA chondrocytes. As shown in Figure 2(c), the extracellular and intracellular areas were both stained with Alizarin Red S at 12 days. Higher levels of calcium deposition were induced by siPTHrP than in the other groups, whilst those in the rhPTHrP group were lower.

### 3.3. PTHrP Is a Direct Target of miR-15a-5p and Is Negatively Correlated with Its Expression in Human Primary Cultured Chondrocytes

**Figure 3:**
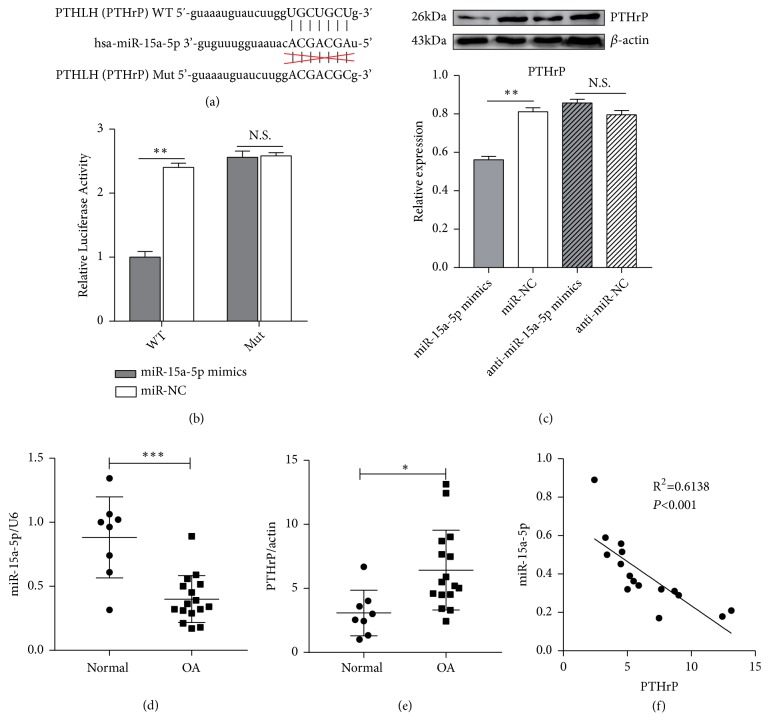
*PTHrP is a direct target of miR-15a-5p*. (a) Sequences of miR-15a-5p and potential miR-15a-5p-binding sites in the 3′UTR of PTHrP. (b) Luciferase activity assay for pGL3-PTHrP-wt or pGL3-PTHrP-mut versus the activity of Renilla luciferase in HEK293T cells after transient transfection with miR-NC or miR-15a-5p mimics (*∗∗p*<0.001). (c) Level of PTHrP in OA chondrocytes treated with miR-15a-5p-mimics or anti-miR-15a-5p by western blot (*∗∗p*<0.001). (d, e) RT-PCR analysis of the relative expression of miR-15a-5p or PTHrP (*∗p*<0.05, *∗∗p*<0.001, n(normal) = 8, and n(OA) = 16). (f) Correlation between miR-15a-5p and PTHrP in OA chondrocytes.

PTHrP 3′UTR contains miR-15a-5p binding sites (Figure 3(a)). To confirm that miR-15a-5p targets PTHrP, a dual-luciferase analysis was performed 48 h after transfection of the HEK293 cells with pmir-PTHrP-wt or pmir-PTHrP-mut reporter vectors. As shown in Figure 3(b), cells cotransfected with miR-15a-5p and the pmiR-PTHrP-wt vector showed a significant decrease in luciferase activity in comparison with the NC group, indicating that miR-15a-5p directly targets PTHrP (*p*<0.01, versus the NC group). Furthermore, the protein levels of PTHrP were analysed by western blotting, which confirmed that miR-15a-5p mimics downregulated the PTHrP protein level (Figure 3(c)). These results confirm that miR-15a-5p can directly bind to the PTHrP mRNA 3′UTR region and regulate the PTHrP protein level, thus indicating that PTHrP is the target for miR-15a-5p.

We used qRT-PCR to measure the levels of miR-15a-5p in normal and OA chondrocytes to determine the expression of miR-15a-5p in human OA chondrocytes. The expression of miR-15a-5p was significantly lower in the OA chondrocytes than in the normal chondrocytes (Figure 3(d)), whilst the expression of PTHrP was statistically higher in the OA chondrocytes than in the normal chondrocytes (*p*<0.05) (Figure 3(e)). A close relationship was observed between miR-15a-5p and PTHrP (*p*<0.01) (Figure 3(f)).

### 3.4. PTHrP Suppresses the Influence of miR-15a-5p on Chondrocyte Proliferation and Terminal Differentiation

**Figure 4:**
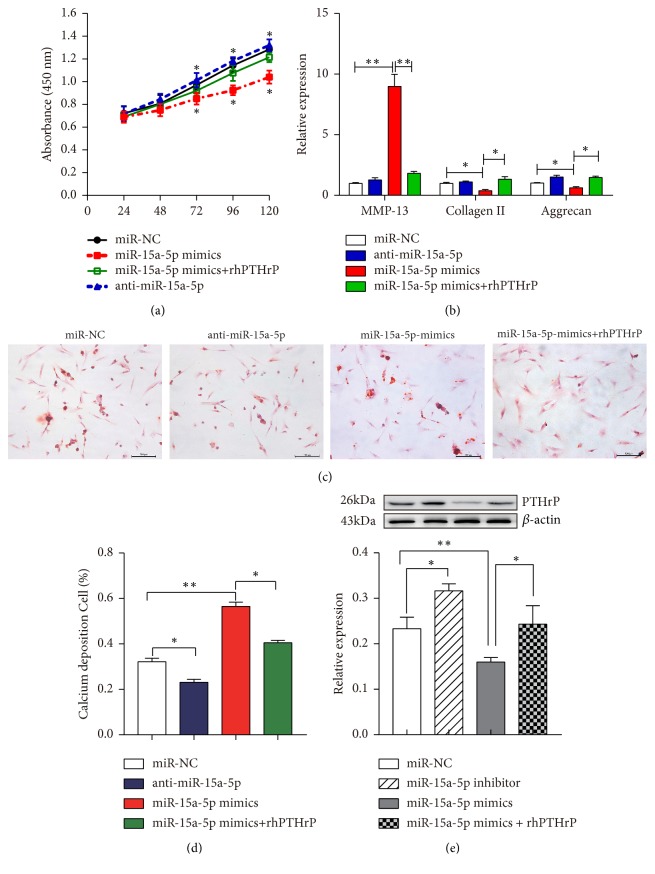
*miR-15a-5p regulates chondrocyte proliferation and differentiation by targeting PTHrP*. OA chondrocytes were treated with miR-15a-5p mimics, miR-15a-5p inhibitors, miR-15a-5p mimics + rhPTHrP, or negative controls. (a) Cell viability was tested with a CCK-8 kit after 24, 48, 72, 96, and 120 h (*∗p*<0.05). (b) RT-PCR was used to analyse the relative expression of collagen II, aggrecan, and MMP-13 in the chondrocytes (*∗p*<0.05, *∗∗p*<0.01). (c) Calcium deposition was visualised by Alizarin Red S staining (scale bars, 100 *μ*m). (d) Calcium deposition-positive cells from Alizarin Red S staining are presented as mean ± SD in the lower panels (*∗p*<0.05, *∗∗p*<0.001). (e) Western blotting analysis of PTHrP expression in transfected cells treated with miR-15a-5p mimics, miR-15a-5p inhibitors, miR-15a-5p mimics + rhPTHrP, or negative controls (*∗∗p*<0.001, *∗p*<0.05, and n = 6).

To understand whether miR-15a-5p has a negative effect on chondrocytes, as shown in Figure 4(a), the CCK8 assay was used to evaluate cell proliferation. The growth rate of primary cultured chondrocytes transfected with miR-15a-5p mimics was slower than that seen in the control group, whilst the growth rate of the miR-15a-5p inhibitors increased slightly.

qRT-PCR was performed to further detect the effects of miR-15a-5p mimics on the proliferation and terminal differentiation of chondrocytes. Chondrocytes were transfected with miR-15a-5p mimics and the inhibitors, and the markers of proliferation and terminal differentiation were investigated, including type II collagen, aggrecan, and MMP-13. Transfection with the miR-15a-5p mimics led to decreases in the expression of collagen II and aggrecan (*p<*0.05) (Figure 4(b)). At the same time, the level of MMP-13 was substantially higher after treatment with the miR-15a-5p mimics than in the control group (*p*<0.01).

Alizarin Red S (ARS) staining at 12 days was also used to identify calcium deposits in the chondrocytes. As shown in Figure 4(c), both the extracellular and intracellular areas of Alizarin Red S staining were observed. The calcium deposition in the miR-15a-5p mimics group was higher than in the other groups, whilst that in the anti-miR-15a-5p group was lower (Figure 4(d)). The level of PTHrP in the chondrocytes increased significantly when endogenous miR-15a-5p was used as a knockdown and, at the same time, the expression of PTHrP decreased when endogenous miR-15a-5p was upregulated via transfection with specific miR-15a-5p mimics or inhibitors. Moreover, the role of miR-15a-5p in regulating the level of PTHrP in the chondrocytes was rescued by the addition of exogenous PTHrP (rhPTHrP) to the cell culture medium (Figure 4(e)).

## 4. Discussion

This study reveals a new mechanism of action of PTHrP in the development of OA. We found that PTHrP was upregulated in OA chondrocytes, which is in agreement with several studies in which the expression of PTHrP in OA cartilage was shown to be higher than in normal human articular cartilage [26, 28]. Several reports also suggested that PTHrP is a vital tumour prognostic factor [29, 30], and a recent study found that PTHrP protected chondrocytes from degeneration [31]. Our function verification analysis also revealed that upregulated PTHrP promoted the proliferation of chondrocytes, whilst the forced downregulation of PTHrP can reduce this effect, which confirms the protective role of PTHrP in suppressing the terminal differentiation of chondrocytes.

Although miRNAs play an important role in the regulation and maintenance of normal physiological conditions, in pathological situations, their levels may change due to dysfunction [14, 32–34]. Consequently, findings regarding the function of miRNAs in OA have varied depending on their expression and the genes targeted [17, 35]. PTHrP has been reported to be a direct target of miR-126-5p in giant cell tumours and of miR-33a in lung cancer [36, 37]. We found that PTHrP was also a potential target of miR-15a-5p in OA. MiR-15a-5p is a member of the miR-15 family, and the expression of miR-15a-5p has been found to be associated with tumour progression, especially in terms of cell proliferation and differentiation [18, 19]. We observed that the expression of miR-15a-5p was downregulated in human OA chondrocytes and was negatively related to the expression of PTHrP. Furthermore, in OA chondrocytes, we found that miR-15a-5p regulated the expression and function of PTHrP.

The levels of collagen II, aggrecan, and MMP-13 in chondrocytes have been found to be dysregulated in degenerative joint diseases including OA, and these abnormal expression patterns can therefore be regarded as biomarkers of chondrocyte function [27, 38]. MMP-13 is reportedly overexpressed in OA, whilst the expression of collagen II and aggrecan is reduced [39]. Aggrecan and collagen II are critical components of cartilage structure and are important for joint function. Consequently, the synthesis and degradation of aggregated proteoglycan and collagen II can reveal their roles in cartilage degradation during joint injury, disease, and aging [40, 41]. In this study, we discovered that the overexpression of miR-15a-5p significantly increased the MMP-13 levels and reduced the levels of type II collagen and aggrecan. This is contrary to the effect of PTHrP. Wang et al. found that PTHrP inhibited the expression of MMP-13 in antler chondrocytes [42], whilst other studies observed that the application of PTHrP significantly increased the expression of type II collagen and aggrecan [31].

Calcium deposition is a marker of chondrocyte terminal differentiation and pathological calcification in osteoarthritic joints [43, 44]. The two most common pathologically relevant calcium crystals deposited in particular tissues are calcium pyrophosphate dihydrate and basic calcium phosphate [45]. Calcium deposition was reported to be closely related to chondrocyte differentiation. In our study, we found that PTHrP reduces calcium deposition. In addition, we found that miR-15a-5p promotes calcium deposition by targeting PTHrP. Our study also showed that miR-15a-5p overexpression may facilitate OA. However, these findings were only observed* in vitro* and will be validated in an animal model in subsequent experiments.

## 5. Conclusions

We demonstrated that miR-15a-5p is downregulated in OA chondrocytes in relation to the upregulated expression of PTHrP. MiR-15a-5p promotes the degeneration of chondrocytes by targeting PTHrP. Our findings provide new evidence that may be useful for future research on targeting miR-15a-5p in the treatment of OA.

## Figures and Tables

**Table 1:** Patients information.

Group	Diagnosis (K-L Grade)	Gender		Age
		Female	Male	
OA	OA (III)	2	0	72.5 ± 3.7
	OA (IV)	8	6	
Normal	Osteosarcoma	3	5	17.6 ± 4.0

Grading standards based on Kellgren and Lawrence system (K-L Grade).

**Table 2:** Primer sequences used in this study.

Name	5′-3′ sequence
PTHrP plasmid	F:CTAGCATTTTGTAAATGTATCTTGGTGCTGCTGAATTTCT ATATTTTTTGTAACATAATGCACTTTAGATATACATATCAA GT
	R:CTAGACTTGATATGTATATCTAAAGTGCATTATGTTACAA AAAATATAGAAATTCAGCAGCACCAAGATACATTTACAAAA TG
PTHrP mutant plasmid	F:CTAGCATTTTGTAAATGTATCTTGGACGACGAGAATTTCT ATATTTTTTGTAACATAATGCACTTTAGATATACATATCAA GT
	R:CTAGACTTGATATGTATATCTAAAGTGCATTATGTTACAA AAAATATAGAAATTCTCGTCGTCCAAGATACATTTACAAAA TG
siPTHrP	F: GGUGGAGACGUACAAAGAGTT
	R: CUCUUUGUACGUCUCCACCTT
miR-15a-5p (mimics)	F: UAGCAGCACAUAAUGGUUUGUG
	R: CAAACCAUUAUGUGCUGCUAUU
miR-15a-5p (control)	F: UUCUCCGAACGUGUCACGUTT
	R: ACGUGACACGUUCGGAGAATT
miR-15a-5p (inhibitors)	F: CACAAACCAUUAUGUGCUGCUA
*β*-Actin	F: CACCCAGCACAATGAAGATCAAGAT
	R: CCAGTTTTTAAATCCTGAGTCAAGC
PTHrP	F: CTGGTTCAGCAGTGGAGCGT
	R: AAGGAAGAATCGTCGCCGTA
U6	F: CTCGCTTCGGCAGCACA
miR-15a-5p	F: GCGTAGCAGCACATAATGGTTTGTG
Collagen II	F: CAGGACCAAAGGGACAGAAAGG
	R: GCAAAGTTTCCACCAAGACCAG
Aggrecan	F: AGCTCTGGGGAGGAATCTGG
	R: GCAGTTCACCAACCGTAGGAGT
MMP-13	F: GCCTTCAAAGTTTGGTCCGATG
	R: TGGTCAAGACCTAAGGAGTGGC

## Data Availability

The data used to support the findings of this study are available from the corresponding author upon request.
